# Knowledge, Attitude, Perceptions, and Concerns of Pregnant Women Regarding the Influenza Vaccination in Kocaeli, Turkey

**DOI:** 10.7759/cureus.23765

**Published:** 2022-04-02

**Authors:** Murat Sağlam, Selim Öncel, Zuhal Gündoğdu

**Affiliations:** 1 Pediatrics and Child Health, Kocaeli University, Izmit, TUR

**Keywords:** pregnant women, vaccination refusal, influenza, vaccination hesitancy, pregnancy, child, turkey, vaccines

## Abstract

Introduction

In order to better understand the barriers to influenza vaccination, we have designed a study to investigate pregnant women's knowledge, attitude, perceptions, and concerns towards the inactivated influenza vaccine.

Materials and methods

In this prospective study, carried out between July 1, 2019, and December 31, 2019, 252 pregnant women (≥12 weeks of gestational age), who had consented to be enrolled in the study, were asked to complete an interviewer-administered questionnaire during their stay in the hospital ward.

Results

The lack of information about influenza vaccines (59.4%) was in the first rank among the reasons for personal and parental vaccine rejection. Most (65.1%) pregnant women either did not believe in or had doubts about the efficiency of the influenza vaccine during gestation. Most of them (80.9%) either were not sure about or did not have much confidence in the prenatally inoculated vaccine's ability to protect their babies postnatally. Some (25.6%) participants attributed their vaccinal unwillingness to fear of adverse effects, and some (11.5%) deemed gestational influenza immunization unnecessary because of their tendency to underestimate the grave consequences of influenza contracted by infants within the first six months of life. The higher the education level, the higher was the knowledge of and the willingness to be immunized against influenza.

Discussion

It is known that about one-third of pregnant women in Turkey have never heard of the influenza vaccine. Unawareness of influenza vaccination in pregnancy is significantly related to vaccine uptake in Japan. Patient education on knowledge of influenza and influenza vaccination has a booster effect on vaccination rates. Had the influenza vaccine been routinely administered to pregnant women in Turkey, the lives of the majority of the women who died of influenza in the influenza A (H1N1) pandemic in 2009 would have been saved. Low-risk perception is quoted as one of the main reasons for influenza vaccine refusal during pregnancy, which was also the case in our study. Concordant with the current literature, the education level and household income were correlated with the level of influenza vaccination uptake. Pregnant women's fear of adverse effects of the influenza vaccine might extend to the fear of birth defects.

Conclusion

The acceptance of influenza vaccines by pregnant women is a consequence of complex interactions of various factors. Giving necessary information to pregnant women is one of the most important factors that can increase influenza vaccine uptake. For this reason, it should be ensured that the efficacy and safety data of the influenza vaccine are more widely included in vocational training activities of health personnel and in prenatal care textbooks and guidelines.

## Introduction

Influenza is a major global health issue with considerable clinical and economic impact. Every year three to five million cases of severe influenza are diagnosed worldwide, 250,000-500,000 of which are subsequently fatal [1].

Pregnant women and their fetuses are vulnerable to the detrimental effects of influenza throughout pregnancy [2]. Premature birth, low birth weight, growth retardation, spontaneous abortion, and other adverse outcomes may occur due to maternal influenza infection during pregnancy [3]. Besides, infants less than six months of age are at significant risk of serious complications from influenza but are too young to be vaccinated [4]. During this period, the best strategy to protect both the mother and fetus (and subsequently the infant) from influenza is to vaccinate the expectant mother [5]. This strategy reduces the risk of influenza infection and the rate of influenza hospitalizations [4].

Since 2004, the American College of Obstetricians and Gynecologists and the Advisory Committee on Immunization Practices have been recommending the seasonal influenza vaccine for all pregnant women throughout the influenza season [6,7]. In 2012, the World Health Organization advised that all pregnant women be vaccinated against influenza [8].

Despite the demonstration of influenza vaccines' clear benefit for pregnant women and their fetuses, vaccination rates continue to fall short of expectations worldwide, mostly because of a lack of communication [2].

This study was conducted to better understand the barriers to influenza vaccination by investigating pregnant women's knowledge, attitude, perceptions, and concerns toward influenza vaccination.

## Materials and methods

Study design and sampling

Information from a convenience sample of 252 pregnant women (≥12 weeks of gestational age) was gathered via a survey during their stay in our university hospital ward between June 1, 2019, and December 31, 2019. Pregnant women having immunological problems, chronic illnesses, risk of premature delivery, hypertension, gestational diabetes, or other pregnancy complications, and fetuses that are small for gestational age, were not included in the study.

Questionnaire design

A questionnaire was designed to reveal the relevant personal, demographic, and social characteristics of the pregnant women and their families.

The questionnaire content was based on the Health Belief Model (HBM), with 15 questions for demographic characteristics and eight questions based on a five-point Likert scale for attitudes towards influenza vaccination. Women indicated their level of agreement with a statement ranging from "strongly agree" to "strongly disagree", which was subsequently converted to an ordinal scale with scores corresponding to one and five, respectively. The survey included questions directed to the level of knowledge about the influenza vaccine and the source of this information. After the completion of the questionnaire, the women were briefed on the influenza vaccine by the interviewer and then were asked to respond to the health belief questions once again.

Statistical analysis

SPSS version 17 (IBM Inc., Armonk, New York) was used for statistical analysis. Kolmogorov-Smirnov test was employed to evaluate the normal distribution. Numerical variables with a normal distribution were given as mean ± standard deviation, while the numerical variables that do not conform to a normal distribution were given as median, and the categorical variables were given as frequency (%). The group differences were tested with the Mann-Whitney U test and the Kruskal-Wallis one-way analysis of variance. Furthermore, Dunn's multiple comparisons were used for numerical variables that do not show a normal distribution. Intervariable relationships were determined by the Spearman correlation. For the testing of two-sided hypotheses, p<0.05 was interpreted as statistically significant.

Ethical considerations

This study was carried out by means of the ethical approval granted by the Kocaeli University Noninterventional Clinical Research Ethics Committee.

## Results

More than half of the questionnaires (n=136) (53.9%) were filled out by women between 25 to 34 years of age. Most participants (n=95) (37.6%) had a gestational age of 30-35 weeks. The numbers of pregnant women who are knowledgeable and unknowledgeable about the influenza vaccine were 136 (53.9%) and 116 (46.0%), respectively. Significant relationships were found between the participants' knowledge about the influenza vaccine and age of the participant, household income, personal and spousal education level, number of live births, place of residence, and personal and spousal profession, whereas gestational age and family type did not seem to play a role (Table 1).

**Table 1:** Demographic factors regarding knowledge about the influenza vaccine ^1^Large city that dominates all the urban and rural communities around the country or region economically. ^2^A residential area where the majority of the population is engaged in trade, industry or management, and there is no agricultural activity. ^3^One of territorial and administrative divisions, into which a province or a metropole is divided

Factors	# of pregnant women, knowledgeable about influenza vaccine (%)	# of pregnant women, not knowledgeable about influenza vaccine (%)	p-value
Age of participant			0.042
18-24	40 (15.9)	25 (9.9)	
25-34	75 (29.7)	61 (24.2)	
35-44	17 (6.7)	28 (11.1)	
45-54	3 (1.2)	2 (0.8)	
55-65	1 (0.4)	0	
Gestational age (in weeks)			0.655
12-17	11 (4.4)	6 (2.4)	
18-23	14 (5.6)	18 (7.1)	
24-29	31 (12.3)	23 (9.1)	
30-35	54 (21.4)	41 (16.3)	
36-41	26 (10.3)	28 (11.1)	
Household income (monthly)			0.001
Low or very low (<1,000₺)	0	0	
Lower-middle (1,000-2,000₺)	4 (1.6)	0	
Middle (2,000-3,000₺)	32 (12.7)	16 (6.3)	
Upper-middle (3,000-5,000₺)	63 (25.0)	49 (19.4)	
High (>5,000₺)	35 (13.9)	51 (20.2)	
Nonrespondents	0	2 (0,8)	
Participant’s education level			<0.001
Without formal schooling	4 (1.6)	3 (1.2)	
Primary education	18 (7.1)	10 (4.0)	
Secondary education	63 (25.0)	41 (16.3)	
University education	50 (19.8)	55 (21.8)	
Master’s study	1 (0.4)	7 (2.8)	
Spousal education level			0.002
Without formal schooling	0	0	
Primary education	6 (2.4)	4 (1.6)	
Secondary education	64 (25.4)	31 (12.3)	
University education	66 (26.2)	71 (28.2)	
Master’s study	0	10 (4.0)	
Number of live births			0.434
0	72 (28.6)	53 (21.0)	
1	47 (18.7)	51 (20.2)	
≥2	17 (6.7)	12 (4.8)	
Place of residence			0.344
Metropole (inner city)^1^	59 (23.4)	51 (20.2)	
Province (urban)^2^	48 (19.0)	51 (20.2)	
District (semirural)^3^	20 (7.9)	12 (4.8)	
Village (rural)	9 (3.6)	2 (0,8)	
Participant’s profession			0.001
Manager	7 (2.8)	5 (2.0)	
Health professional	11 (4.4)	10 (4.0)	
Other highly-skilled worker	11 (4.4)	10 (4.0)	
Middle-skilled worker	36 (14.3)	29 (11.5)	
Housewife	67 (26.6)	66 (26.2)	
Spousal profession			0.003
Manager	12 (4.8)	9 (3.6)	
Health professional	3 (1.2)	19 (7.5)	
Other highly-skilled worker	17 (6.7)	17 (6.7)	
Middle-skilled worker	72 (28.6)	48 (19.0)	
Unemployed	34 (13.5)	21 (8.3)	
Family type			0.674
Nuclear	110 (43.7%)	92 (36.5)	
Extended	24 (9.5%)	21 (8.3)	
Single-parent	2 (0.8%)	3 (1.2)	

The pregnant women's source of knowledge on influenza vaccine was mostly health professionals (Table 2).

**Table 2:** The pregnant women's source of knowledge on the influenza vaccine

Source	# of pregnant women (%)
Health professionals	58 (42.6%)
Mass media	52 (38.2%)
Friends	26 (19.1%)

Fifty-seven (22.6%) participants were willing to be immunized with the influenza vaccine for the benefit of themselves and their unborn babies. For the remaining 195 (77.4%), the reported reasons behind the reluctance or rejection of influenza vaccination are shown in Table 3. Lack of information about the influenza vaccine was in the first rank among the reasons for vaccine rejection.

**Table 3:** Main reasons behind maternal rejection of the influenza vaccine

Main reason	# of pregnant women reporting the reason (%)
Financial insufficiency	4 (2.0%)
Lack of time	20 (10.2 %)
Lack of knowledge	116 (59.4%)
Fear of adverse reactions	50 (25.6%)
Contracting flu before the vaccine for that flu season comes on the market	5 (2.5%)

Similarly, with their tendency to reject the influenza vaccine, the majority of the pregnant women (n=213) (84.6%) did not accept that their children should be immunized against influenza when they are old enough (older than six months of age).

Most pregnant women had doubts about the efficiency of influenza vaccines, such that 28 (11.1%) women disagreed, and 136 (54.0%) were not sure that the influenza vaccine would protect them against influenza during pregnancy. When it came to protecting the fetus and, subsequently, the infant with the vaccine inoculated during pregnancy, disagreement and uncertainty was noted in 48 (19.0%) and 156 (61.9%) participants, respectively.

Responses to other HBM statements are shown as mean (SD) and median scores on a five-point Likert scale in Table 4.

**Table 4:** Responses of pregnant women to Health Belief Model statements regarding the influenza vaccine

Statements	Mean (standard deviation)	Median score
"Influenza vaccine prevents the infant from getting influenza in the first six months of life" (perceived benefit)	3.0 (0.70)	3
"My child may get influenza if not immunized" (perceived benefit)	2.93 (0.67)	3
"Influenza vaccine prevents absenteeism in the workplace" (perceived benefit)	3.24 (0.72)	3
"Influenza vaccine prevents influenza complications" (perceived severity)	3.04 (0.74)	3
"Getting all flu shots is important for my child's health" (perceived severity)	3.76 (0.74)	4
"Vaccine is worthwhile even if its immunity does not last for life" (perceived severity)	3.14 (0.76)	3
"Since influenza is a minor illness even for infants, being immunized against it for the benefit of my baby seems unnecessary" (perceived severity)	2.77 (0.69)	3
"Risks of influenza vaccine outweigh its potential benefits" (perceived barrier)	3.68 (0.90)	4
"I am comfortable with the number of shots my child will receive" (perceived barrier)	3.63 (0.81)	4

In the second part of this study, the pregnant women were given information on influenza vaccination, after which they were once again questioned about their willingness to be and get their infants vaccinated. After an informative intervention, the number of women accepting the vaccine increased from 57 (22.6%) to 103 (40.9%).

## Discussion

Vaccination uptake

Our results showed that approximately half of the pregnant women (46.0%) were not aware of the existence of an influenza vaccine. In a cross-sectional study carried out with 786 participants in Northern Turkey in 2016, 31.6% of pregnant women had never heard of the influenza vaccine [9]. In some countries, the most important obstacle to influenza vaccination is lack of knowledge: in a study conducted in Japan, unawareness of influenza vaccination in pregnancy (13%), although much lower than in Turkey, was significantly related to vaccine uptake, which gives rise to the thought that it is probably true for Turkey [10].

Healthcare professionals are a reliable and the foremost (42.6% in our study) source of information about influenza vaccination. Their provision of health information to expectant mothers actually serves to increase the acceptance level of maternal and filial influenza vaccination (an 18.3% increase in this study). Yudin et al. demonstrated the impact of patient education on knowledge of influenza and influenza vaccination in their study by obtaining an even more striking increase (37%) in vaccination rates within one year [11].

Perceived benefits of vaccination

In an Australian study, significant proportions of women believed that the flu vaccine would not have a protective effect on them (30%) or their babies (26%) [12]. Similar results were seen in a United States study such that 25% of unvaccinated pregnant women did not find the influenza vaccine effective [13]. However, in a randomized-controlled study from Bangladesh, like in many others, the frequency of influenza-like illness was shown to have decreased in the mothers and their infants by means of immunizing the pregnant women by 36% and 29%, respectively [14]. In Turkey, 656 lives were officially reported to have been lost during the influenza A (H1N1) pandemic in 2009, of which 41 (6.3%) were pregnant or puerperal women [15]. Had the influenza vaccine been routinely administered to pregnant women in Turkey, the majority (63%) of these 41 and unreported many others would not have fallen victim to a vaccine-preventable disease like influenza [14].

The awareness of the secondary benefits of the influenza vaccine, along with its clear primary protective effect, was very low in the pregnant women participating in our study. However, with the implementation of influenza vaccination, workplace absenteeism is reduced, and time and health expenditures can be saved. In a study conducted in England and Wales, the incremental cost-effectiveness ratio is £23,000 per quality-adjusted life-year gained [16].

Perceived severity of influenza infection

Most (77.4%) of the pregnant women participating in our study were skeptical about the necessity of the influenza vaccine because a considerable proportion (11,5%) of them agreed with the statement that influenza was a minor illness in infants (a mean of 2.77 Likert-scale points) (median score=3). In a study conducted among obstetrician-gynecologists in Italy, low risk perception (82.3%) was quoted as the main reason for influenza vaccine refusal during pregnancy [17].

Parents are inclined to take a more cautious attitude towards overtly dangerous and detrimental illnesses such as invasive meningococcal disease, which often gives way to a high willingness to have their children vaccinated. In a Canadian study, the parental acceptance rate of serogroup B meningococcal vaccine was found to be 93%, which is in sharp contrast to the acceptance rate of 22.6% found for inactivated influenza vaccine in our study [18].

Confirming the hypothesis of "the higher the education level, the higher the vaccine uptake" in general, our study showed that this hypothesis was also true for the influenza vaccine [18-21]. Concordant with previous research on other vaccines, our results show that household income is correlated with influenza vaccination uptake [22].

Perceived barriers to vaccination

The necessity of repeating vaccination annually in order to stay protected against influenza inevitably seems to have created a perception of barriers and a high level of discomfort among the pregnant women taking part in our study (median score=4). Injection phobia was denoted as a reason for refusal of the influenza vaccine in the systematic review, including 45 studies by Yuen et al. [23].

Our survey revealed that the majority of pregnant women did not want to be vaccinated because of their fear of adverse effects of the influenza vaccine (median score=4). In the meta-analysis by Yuen et al., the first reason for pregnant women not getting immunized was the concern about the potential adverse effects of the vaccine [23]. In a single-center study conducted in Toronto, Canada, the results showed that 21% of the pregnant women believed that studies have shown the vaccine could cause birth defects [24].

Study strengths and limitations

As a strength, this is one of a few studies conducted in Turkey to reflect the attitudes of pregnant women toward influenza vaccination [9,25-28]. It provides useful information for national and foreign health policymakers on the current status of and the factors related to pregnant women's personal and parental hesitancy on influenza vaccination in Turkey and to make comparisons.

A limitation of the study is that it was conducted in a single tertiary health center. However, this is mitigated to a certain extent by the fact that Kocaeli is one of the most industrialized and cosmopolitan provinces in Turkey; therefore, our participants probably constitute a balanced study population from all walks of life. Another limitation may involve the cross-sectional type of the study - the answers were self-reported. Other limitations are the usage of an unvalidated questionnaire and a nonrepresentative sample of all Turkish pregnant women. Additionally, in a questionnaire-based study like this, social desirability bias may have played a role, i.e., participants may have given answers to meet the expectations of their families or colleagues.

## Conclusions

The acceptance of influenza vaccines by pregnant women is the result of complex interactions of various factors. Inadequate knowledge about the consequences of influenza, uncertainty about vaccine safety, efficacy, and benefits are major barriers to vaccination. Our findings underline a high prevalence of rejection or, at least, hesitancy among pregnant women regarding getting and having their infants immunized against influenza, which means putting up a wall against protection from the complications of this potentially-severe disease during gestation and infancy, especially in countries where influenza vaccine has not yet been incorporated in the national immunization program. It is also noteworthy that even a short informative talk with pregnant women by an interviewer has a positive effect on influenza vaccine acceptance. Aware of this great power in their hands, healthcare professionals should capture every opportunity to popularize the influenza vaccine by informing their patients about its safety and efficacy. This issue should be given importance in the vocational training activities of healthcare professionals. In addition, it is important that the efficacy and safety data of influenza vaccine have a wider place in prenatal care textbooks and guidelines.
